# Liberal versus conservative oxygen therapy in critically ill patients: using the fragility index to determine robust results

**DOI:** 10.1186/s13054-018-2165-z

**Published:** 2019-04-18

**Authors:** Maria Vargas, Giuseppe Servillo

**Affiliations:** Department of Neurosciences, Reproductive and Odontostomatological Sciences, University of Naples “Federico II”, via Pansini, 80100 Naples, Italy

Guidelines on the use of supplemental oxygen for various acute illnesses in adults are contradictory and inconsistent, and at this time no high-quality evidence base exists [1]. Although many randomized controlled trials (RCTs) comparing liberal versus conservative oxygen for various critical conditions have been done, the trial data are not conclusive [2]. With this in mind, we read with great interest the just published systematic review and meta-analysis by Chu et al. [2] on mortality and morbidity in acutely ill adults treated with liberal versus conservative oxygen therapy. The authors stated that liberal supplemental oxygen was harmful and associated with a dose-dependent increased risk of short-term and long-term mortality [2]. Chu et al. performed an excellent statistical analysis and a robust trial sequential analysis. Our major concern is about the fragility of the included trials. As we know from the current literature, RCTs are designed to assess objectively the safety and efficacy of a specific intervention [3]. Recently the fragility index (FI), an intuitive measure of the robustness of RCTs, was introduced in the critical care area [4]. Studies with larger FI values have more robust findings compared with the studies with poor FI values [3, 4]. We evaluated the FI of the RCTs included in the meta-analysis by Chu et al. using a two-by-two contingency table and *p* value produced by Fisher exact test [4]. Surprisingly, we found that 18 RCTs had a FI of 0 while the unpublished data from trial NCT00414726 had a FI of 2 (Table 1). Furthermore, we looked at the statistical significance of the mortality reported in the included study and found that only the study by Girardis et al. [5] reached statistical significance in the primary outcome.

**Table 1:** Fragility index calculated for the study included in the systematic review and meta-analysis by Chu et al. [1]

Study	Setting	Liberal group (FiO2 0.52 CI:0.39-0.85)	Conservative group (FiO2 0.21 CI:0.21-0.50)	*p* value for mortality in each study	Fragility index n (p)
Ali et al. (2014) [6]	Stroke	5/155	4/146	0.99	0 (1)
Roffe et al. (2017) [7]	Stroke	50/2668	45/2668	0.75	0 (0.506)
Ronning et al. (1999) [8]	Stroke	36/292	27/258	0.54	0 (0.506)
Singhal et al. (2005) [9]	Stroke	0/9	1/7	NS	0 (0.438)
NCT00414726	Stroke	14/43	4/42	0.08	2 (0.072)
Shi et al. (2017) [10]	Stroke	0/9	0/9	NS	0 (1)
NCT02378454 (2015)	Sepsis	3/25	2/25	NS	0 (1)
Butler et al. (1987) [11]	Limb ischemia	1/17	0/22	NS	0 (0.436)
Schietroma et al. (2016) [12]	Perforated peptic ulcer	2/119	4/120	0.42	0 (0648)
NCT02687217	Acute appendicitis	0/30	0/30	NS	0 (1)
Girardis et al. (2016) [5]	Critical illness	80/243	58/235	0.01	0 (0.055)
Panwar et al. (2016) [13]	Critical illness	12/51	13/53	0.44	0 (1)
Hofmann et al. (2017) [14]	Myocardial infarction	53/3311	44/3318	0.08	0 (0.359)
Khoshnood et al. (2015) [15]	Myocardial infarction	3/85	3/75	NS	0 (1)
Kuisma et al. (2006) [16]	Cardiac arrest	4/14	4/14	NS	0 (1)
Rawles et al. (1976) [17]	Myocardial infarction	9/105	3/95	NS	0 (0.140)
Stub et al. (2012) [18]	Myocardial infarction	5/132	11/132	NS	0 (0.204)
Ukholkina et al. (2005) [19]	Myocardial infarction	1/58	0/79	NS	0 (0.428)
Young et al. (2014) [20]	Cardiac arrest	5/9	4/8	NS	0 (1)

*NS* not significant

The authors stated that their data may have potential implications in clinical practice of acutely ill patients. According to our results, critical care clinicians should be wary of basing decisions about conservative or liberal oxygen therapy on the available information from this meta-analysis [1] including trials with a low FI.

Furthermore, these results came from fragile and not statistically significant RCTs. Maybe it is time to add the FI and/or statistical significance of the considered outcome as criteria for the evaluation of the quality of evidence.

## Abbreviations

FI: Fragility index
RCTs: Randomized controlled trials
